# Impacts of leukocyte filtration and irradiation on coagulation factors in fresh frozen plasma

**DOI:** 10.3892/etm.2014.2126

**Published:** 2014-12-10

**Authors:** DAI-YU LI, HONG-WEI ZHANG, QING-ZHEN FENG, HUA ZHAO

**Affiliations:** 1 Department of Blood Transfusion, The Affiliated Hospital of Luzhou Medical College, Luzhou, Sichuan 646000, P.R. China; 2 Department of Clinical Laboratory, The Affiliated Hospital of Luzhou Medical College, Luzhou, Sichuan 646000, P.R. China

**Keywords:** leukocyte filtration, irradiation, fresh frozen plasma, coagulation system, coagulation factor

## Abstract

This study aimed to compare and analyze the changes in the coagulation factors in fresh frozen plasma (FFP) prior to and following leukocyte filtration and irradiation. In total, 30 bags of FFP from healthy donors were processed: One-third of the FFP of each bag was left within the original bag (the A group), the other two-thirds of the FFP of each bag were passed through a disposable leukocyte filter, then divided equally into two parts. One of these was designated as the B group, and the other was designated the C group (subjected to 30 Gy irradiation). All samples were analyzed to evaluate 16 coagulation indicators. Analysis of variance revealed that there were statistically significant differences in the levels of fibrinogen (FbgC) and coagulation factor VIII (FVIII:C) among the groups (P=0.044 and P=0.015, respectively); the Dunnett’s t-test revealed that there was a statistically significant difference in the level of FbgC between the A and B groups (P=0.025), and there was a statistically significant difference in the level of FVIII:C between the A and C groups (P=0.009); while the remaining 14 coagulation parameters were not significantly different among the groups. Although the levels of FbgC and FVIII:C in the FFP were reduced following treatment, this would not affect the clinical effect of the FFP.

## Introduction

Fresh frozen plasma (FFP) is an alternative therapy mainly used in the treatment of clinical bleeding, which occurs due to the deficiency of single and multiple coagulation factors induced by various causes. Leukocytes are a non-therapeutic component of FFP, which act as a kind of pollutant and increase the risk of adverse transfusion reactions when performing allogeneic transfusion. Currently, the main method used to clear the plasma of residual leukocytes is leukocyte filtration. Removal of leukocytes by this method can significantly reduce adverse transfusion reactions; however, a certain number of leukocytes remain in the FFP, and some of these have the ability to proliferate (1,2). The leukocyte filtration method used alone cannot sufficiently prevent the occurrence of transfusion-associated graft-versus-host disease (TA-GVHD) (2,3). Although cases of FFP-infusion-induced TA-GVHD are rare there have been some reported cases in which, following filtration 10^6^–10^8^ lymphocytes remain in the FFP, and these remaining lymphocytes could cause TA-GVHD when used by patients with immune function defects or suppression, or by patients who have undergone bone marrow transplantation (4). This study investigated the coagulation factors that remained in FFP following leukocyte removal and irradiation, with the aim of determining which coagulation factors were affected, as well as the degree of the effect. This information may be useful in improving the safety and effectiveness of clinical transfusions.

## Materials and methods

### Materials

Thirty bags of FFP, with 50 ml in each bag, were collected from 30 healthy donors by Luzhou city blood bank (Luzhou, China).

Disposable FTS-RC202 (2×200) leukocyte filtration-transfusion equipment was obtained from Nanjing Shuangwei Biotechnology Co., Ltd. (Nanjing, China). This study was conducted in accordance with the Declaration of Helsinki. and with approval from the Ethics Committee of Luzhou Medical College (Luzhou, China). Written informed consent was obtained from all participants.

### Experimental procedure

#### Leukocyte filtration of FFP

The 30 bags of FFP, with 50 ml in each bag, were stored at −20°C. The experiment was conducted five times. Six bags of FFP were taken out from the refrigerator, and after thawing in a frozen plasma thawing box, each bag of FFP was processed using the disposable leukocyte filter and the filtered FFP was equally divided into two bags. The original bag retained 5 ml unfiltered FFP and was designated as the control (the A group). For the two bags of filtered FFP, one was designated the B group (leukocyte removal group), and the other was subjected to irradiation, and designated the C group (leukocyte removal + irradiation group).

#### Plasma irradiation of the C group:

A blood irradiation instrument (Gammacell 1000; MDS Nordion, Ottawa, Canada) was used, with a radiation dose of 30 Gy and irradiation time of 11 min and 50 sec.

#### Detection of coagulation indicators

A CA7000 automated blood coagulation analyzer (Sysmex Corporation, Kobe, Japan) was used to analyze the samples, and the following coagulation factors were tested: prothrombin time (PT; solidification method, Thromborel S Reagent); activated partial thromboplastin time (APTT; solidification method, Dade Actin activated Cephaloplastin Reagent); fibrinogen (FbgC; Dade Thrombin Reagent); thrombin time (TT; solidification method, Test Thrombin Reagent); D-dimer (D-Di; immunoturbidimetry); endogenous coagulation factors VIII, IV and VI (FVIII:C, FIV:C and FVI:C; solidification method, coagulation Factor VIII, IV and VI Deficient Plasma); antithrombin III (AT III; chromogenic substrate method, Berichrom Antithrombin III); and exogenous coagulation factors FII:C, FVII:C, FX:C (solidification method, Coagulation Factor II, VII and X Deficient Plasma). The above reagents were manufactured by Siemens (Marburg, Germany). The fibrin/fibrinogen degradation products (FDPs) were analyzed using the Nanopia P-FDP kit (Sekisui Medical Co., Ltd., Tokyo, Japan).

Sample testing was operated strictly in accordance with the instructions of the instruments and reagents. Quality control testing was firstly performed, followed by sample testing. Following the testing, the results were recorded for analysis.

### Statistical analysis

All data were subjected to statistical analysis with SPSS version 17.0 statistical software (SPSS, Inc., Chicago, IL, USA), and the results are expressed as mean ± standard deviation (SD). The Kolmogorov-Smirnov test was used for normality testing, and Levene’s test was used to evaluate the homogeneity of variance. The randomized block design analysis of variance (ANOVA) was applied, and further pairwise comparisons used the Dunnett’s t-test, with P=0.05 set as the test standard, and P<0.05 was considered to indicate statistically significant difference.

## Results

### FFP analysis results

The results of the three groups fitted a normal distribution and homogeneity of variance, thus meeting the conditions of ANOVA. In the randomized block design ANOVA for intergroup analysis of FbgC, P=0.044, indicating that the values of FbgC among the three groups differed. The Dunnett’s t-test was further performed for pairwise comparison between the experimental groups, and the results revealed that the FbgC contents of the A and B groups were 2.287±0.358 and 1.968±0.296 (P=0.025), respectively. Thus, the difference was statistically significant, that is, following the removal of leukocytes, the FbgC content of FFP was decreased by 14%. The ANOVA result for FVIII:C among the experimental groups was P=0.015, indicating that the values of FVIII:C among the three groups differed. The Dunnett’s t-test was further used for pairwise comparison among the experimental groups, and the results for the A and C groups (mean ± SD, 105.8±49.6 and 87.4±37.5, respectively) were significantly different (P=0.009); that is, FVIII:C was impacted by leukocyte removal and irradiation, and decreased by 17%. ANOVA of the remaining indicators indicated that there were no significant differences between the three groups (P>0.05).

### Trends in the changes of plasma PT, PT%, prothrombin time ratio (PTR), international normalized ratio (INR), APTT and TT

Following leukocyte removal, PT, PTR, INR and APTT were slightly reduced, while PT% and TT exhibited slight increases. Following the irradiation treatment, PT, PTR and INR exhibited a further tendency to reduction, while PT% and APTT showed an increasing trend, and TT slightly decreased. The results are shown in Table I.

### Trends in the changes of endogenous coagulation factor activities

Following leukocyte removal, the activities of FVIII:C and FIV:C decreased slightly, while that of FVI:C tended to increase. Following irradiation, the activities of all the factors tended to decrease. The results are shown in Table II.

### Trends in the changes of extrinsic coagulation factors

Following leukocyte removal, the levels of FII:C, FVII:C and FX:C increased slightly; and following irradiation, FII:C tended to increase whereas FVII:C and FX:C tended to decrease. Statistical analysis indicated that there were no significant differences among the groups. The results are shown in Table III.

### Trends in the changes of AT-III

Following leukocyte removal, the AT-III activity was slightly elevated, and following irradiation, the activity continued to exhibit an increasing trend. The results are shown in Table IV.

### Trends in the changes of FbgC

Following the removal of leukocytes, the FbgC content decreased; however, following irradiation, the FbgC content increased again. The results are shown in Table V.

### Trends in the changes of plasma fibrinolytic indices

Following leukocyte removal, the contents of FDP and D-Di were slightly reduced. Following irradiation, the content of FDP was slightly reduced, and the content of D-Di was slightly increased. The results are shown in Table VI.

## Discussion

FFP is an important component of apheresis, which has a content of coagulation factors higher than that of normal frozen plasma, and is mainly used in the treatment of patients who have a complex deficiency of coagulation factors. Residual leukocytes in FFP increase the risk of transfusion-related infections, such as with certain viruses that have a high affinity with leukocytes. Cytomegalovirus, human immunodeficiency virus, human T-lymphotropic virus and Cretzfeldt-Jakob disease virus all use leukocytes as a carrier or spreading medium, thus resulting in transfusion-related infectious diseases. In addition, a certain amount of allogeneic leukocyte infusion causes various adverse reactions of transfusion (1–4). Therefore, new technologies for transfusion, such as leukocyte filtration and blood irradiation, are particularly important in the clinical application of FFP.

The removal of residual leukocytes from FFP would greatly reduce the risks of transfusion, for example, by reducing febrile non-hemolytic transfusion reaction (FNHTR), reducing the risk of infection by leukocyte-associated viruses such as cytomegalovirus, reducing postoperative infection, the recurrence and metastasis of tumors and transfusion-associated immunosuppression (5,6), and by reducing the incidence of TRALI (7,8). Leukocyte removal is particularly necessary for patients who require multiple transfusions, and could reduce the alloimmunization and inefficiency of platelet transfusion.

A previous study demonstrated that most leukocyte filters are able to filter out ~10^3^–10^4^ leukocytes. It has been reported that 4×10^4^ lymphocytes (normal status: 0.8–4×10^9^) caused patients with severe combined immunodeficiency to experience GVHD (9). In another study, it was reported that the infusion of 10^4^ leukocytes per kg body weight caused the occurrence of GVHD (10). Therefore, the treatment of blood products by leukocyte removal alone is insufficient; γ-ray irradiation to inactivate the lymphocytes remaining in the blood products is also required, as this has been preliminarily exhibited effects in TA-GVHD prevention (11). Therefore, these two common methods, namely the removal and inactivation of leukocytes, have an important role in clinical transfusion; the clinical application of these technologies could prevent and avoid the aforementioned adverse transfusion reactions and complications. The clinical application of FFP is normally connected with the supplementation of coagulation factors or the correction of coagulation factor disorders; therefore, it is necessary to consider whether these two methods used together would affect the coagulation factors of FFP.

Domestic and international studies have shown that leukocyte removal can cause the activity of coagulation factors in FFP, such as FVIII and FV, to decrease (12–18), in addition to significantly reducing the incidence of adverse transfusion reactions (19). The impact of leukocyte removal towards the coagulation system would usually be negligible.

In the present study, 30 samples of FFP were collected and subjected to no treatment or to leukocyte removal with or without radiation, and the differences in the coagulation factors in the three groups were determined. A total of 16 coagulation indicators were detected. With the exception of FbgC and FVIII:C, which exhibited some differences from their levels prior to treatment, the remaining indices were not statistically affected.

FbgC, also known as coagulation factor I, is the most abundant and important plasma coagulation factor, with a normal plasma concentration of 2–4 g/l. In this study, FbgC was measured by the Clauss method. For the A, B and C groups, the FbgC levels were 2.287±0.358, 1.968±0.296 and 2.122±0.331 g/l, respectively. That is, there was a 14% reduction of the FbgC level following leukocyte removal, and an increase of 8% following irradiation. ANOVA revealed that the differences among the FbgC levels in the experimental groups had statistical significance (P=0.044). The Dunnett’s t-test conducted for intergroup comparisons, revealed that the difference between the A and B groups was statistically significant (P=0.025), while that between the A and C group was not statistically significant. Following leukocyte removal, the FbgC level in the FFP declined by 14%, while it increased after the irradiation; the magnitudes of these changes were not large, and normally would not affect the clinical use of FFP. It has been reported that a large dose of irradiation (50 kGy) reduces fibrinogen levels (20), while a small dose of radiation may elevate the fibrinogen levels.

FVIII:C is a plasma globulin, which has been termed the antihemophilic factor or antihemophilic globulin. It is very unstable, and its function is to act as the cofactor of FIV:C, and participate the activation of FX:C by FIV:C. The plasma level of FVIII:C is ~0.1 mg/l, the lowest among all the plasma coagulation factors. The results of this study demonstrated the reduction of its content following leukocyte removal and irradiation, with levels of 105.8±49.6, 98.3±44.8 and 87.4±37.5% in groups A, B and C, respectively. Following leukocyte removal, the level of FVIII: C was reduced by 7%, and a further reduction of 11% was observed after irradiation. ANOVA revealed that the differences among the experimental groups were statistically significant (P=0.015), and the Dunnett’s t-test for intergroup comparison further revealed that the difference in values between the A and C groups was statistically significant (P=0.009). The results of this experiment showed that FFP exhibited a reduction of FVIII:C content following the leukocyte removal and irradiation treatments, but the difference between the A and B groups was not statistically significant. The remaining 14 indices of the coagulation system did not show a statistically significant difference between groups; following the leukocyte removal and irradiation treatments; FIV:C and FVI:C were not affected. The PT test is the commonly clinically used screening test of the extrinsic coagulation system. FII:C, FVII:C and FX:C are extrinsic coagulation factors. The PT results for groups A, B and C were 12.4±0.92, 12.0±0.73 and 11.8±0.69, respectively (P=0.089). Concerning the amplitudes of the changes in PT, the PT of the B group was 3% shorter than that of the A group and the PT of the C group was 2% shorter than that of the B group. Thus, it could be observed that leukocyte removal and irradiation both shortened the PT. The change trends of PTR and INR were the same as those for PT, while the PT% increased after processing, indicating that leukocyte removal and irradiation might have activated the extrinsic coagulation system to a certain extent.

Concerning the amplitudes of the changes in FII:C, the FII:C level in the B group was 2% higher than that in the A group, and the FII:C level in the C group was 4% higher than that in the B group. For FVII:C, the level in the B group was 3% higher than that in the A group, and the level in the C group was 2% lower than that in the B group. The FX:C level in the B Group was 13% higher than that in the A group, and the FX:C level of the C group was 1% lower than that of the B group. Based on the above results, after leukocyte removal and irradiation, PT tended to decrease, and the levels of FII:C, FVII:C and FX:C tended to increase after removing the leukocytes; while FII:C increased further after irradiation, FVII:C and FX:C were reduced to a certain extent, although compared with untreated FFP, they were elevated. This indicates that leukocyte removal and irradiation might exert some degree of activation towards the extrinsic coagulation system.

TT is a detection index of anticoagulation and fibrinolysis, and an extension of this is observed as a reduction in the levels of fibrinogen or an increase in the levels of fibrinolytic and anticoagulant products. Following leukocyte removal, the TT was extended by 2%, and following the irradiation treatment, the TT was shortened by 1%. Thus, TT tended to be prolonged after leukocyte removal, and shortened after irradiation; the impact of leukocyte removal and irradiation towards TT exhibited a lengthening trend relative to FFP. Such a trend was consistent with the trend in FbgC levels, while inconsistent with the trend in fibrinolytic product levels. This might be because the amount of fibrinolytic products was very low, and thus the trend could not be observed.

The normal plasma level of AT-III antigen is 80–300 mg/l. Its deficiency can lead to thrombotic disease. In the present study, compared with the AT-III antigen level in the A group, that in the B group was increased by 3%, while there was no difference between the C and B groups.

There have been inconsistent reports concerning the impacts of leukocyte filtration and irradiation towards coagulation factors in FFP. In two studies concerning the impacts of leukocyte removal on coagulation factors, the results differed when the leukocyte filter (LPS1) was used; one of the studies found that the levels of factor VIII, IV and VI did not decline after its use (20), while the other reported that the levels of factor VIII, IV and VI decreased (0–21%) (21).

A previous study of the changes in the coagulation system when leukocyte-removed FFP was subjected to 30 Gy γ-ray irradiation (22), had different results from the present study. In the previous study, the statistically significant changes were that the PT, APTT and FVIII:C levels were reduced, and with an increase in radiation doses, APTT exhibited a U-shaped trend, that is, it shortened initially but then was extended. In addition, the procoagulant activities of factors II, VII, VI and X were increased, and activation of the fibrinolytic system was exhibited. In the present study, the above indices between the B and C group had no statistical significance, among which PT and FVIII:C activities tended to decrease, while the median of APTT was prolonged; FII:C levels tended to increase, the procoagulant activities of factors VII, VI and X were reduced; and FDP and D-Di, the fibrin degradation products, had no tendency to increase.

The leukocyte removal and radiation treatment of FFP may reduce adverse transfusion reactions and prevent TA-GVHD without affecting the activities of the majority of coagulation factors. Although the levels of fibrinogen and FVIII;C exhibited certain reductions, this would not be expected to affect the clinical effects of FFP.

## Figures and Tables

**Table I:** Results of global coagulation tests.

Index	Group A	Group B	Group C
PT (sec)	12.40±0.92	12.01±0.73	11.82±0.69
PT (%)	77.41±13.90	81.39±11.21	84.43±11.68
PTR (%)	1.06±0.08	1.03±0.06	1.01±0.06
INR	1.05±0.07	1.03±0.06	1.01±0.06
APTT (sec)	33.60±5.70	32.81±6.25	33.42±6.02
TT (sec)	20.70±1.43	21.23±1.37	21.04±1.26

Values are mean ± standard deviation (n=10 per group). PT, prothrombin time; PTR, PR ratio; international normalized ratio; APTT, activated partial thromboplastin time; TT, thrombin time. Group A, control; group B, leukocyte removal; group C, leukocyte removal + irradiation. For intergroup comparison of each index, P>0.05.

**Table II:** Intrinsic coagulation factors (%).

Index	Group A	Group B	Group C
FVIII:C	105.8±49.6	98.3±44.8	87.4±37.5
FIV:C	88.6±20.9	85.4±21.1	82.0±14.1
FVI:C	85.9±37.3	89.7±36.2	88.7±35.1

Values presented are mean ± standard deviation (n=10 per group). Group A, control; group B, leukocyte removal; group C, leukocyte removal + irradiation. F, factor. In the intergroup analysis of variance for FVIII:C, P=0.015; in the Dunnett’s t-test comparison between the A and C groups, P=0.009. In the intergroup comparison of the remaining detection indices, P>0.05.

**Table III:** Extrinsic coagulation factors (%).

Index	Group A	Group B	Group C
FII:C	117.6±19.4	119.8±20.6	124.0±21.5
FVII:C	166.4±17.7	171.2±17.5	167.9±15.9
FX:C	129.8±29.4	146.6±23.3	144.7±23.1

Values presented are mean ± standard deviation (n=10 per group). Group A, control; group B, leukocyte removal; group C, leukocyte removal + irradiation. In the intergroup comparison of each detection index, P>0.05.

**Table IV:** AT III activity (%).

Index	Group A	Group B	Group C
AT III-A	88.9±16.6	92.0±11.5	92.2±14.3

Values presented are mean ± standard deviation (n=10 per group). Group A, control; group B, leukocyte removal; group C, leukocyte removal + irradiation. AT III, antithrombin III. In the intergroup comparison of each detection index, P>0.05.

**Table V:** FbgC levels (g/l).

Index	Group A	Group B	Group C
FbgC	2.287±0.358	1.968±0.296	2.122±0.331

Values presented are mean ± standard deviation (n=10 per group). Group A, control; group B, leukocyte removal; group C, leukocyte removal + irradiation. FbgC, fibrinogen. In the intergroup analysis of variance of FbgC (Clauss method), P=0.044. In the Dunnett’s t-test for groups A and B, P=0.025.

**Table VI:** Fibrinolysis indices (μg/ml).

Index	Group A	Group B	Group C
FDP	0.71±0.32	0.68±0.29	0.60±0.28
D-Di	0.091±0.182	0.088±0.172	0.089±0.173

Values are mean ± standard deviation (n=10 per group). Group A, control; group B, leukocyte removal; group C, leukocyte removal + irradiation. FDP, fibrinogen degradation product; D-Di, D-dimer. In the intergroup comparison of each detection index, P>0.05.
